# Increased levels of the inflammatory biomarker C-reactive protein at baseline are associated with childhood sickle cell vasocclusive crises

**DOI:** 10.1111/j.1365-2141.2009.08013.x

**Published:** 2010-03

**Authors:** Suba Krishnan, Yamaja Setty, Suhita G Betal, Vaidyula Vijender, Koneti Rao, Carlton Dampier, Marie Stuart

**Affiliations:** 1 Marian Anderson Comprehensive Sickle Cell Anemia Care and Research Center, Department of Pediatrics, Division of Research Hematology, Jefferson Medical College, Thomas Jefferson UniversityPhiladelphia, PA; 2 Thrombosis Research Center, Temple University School of MedicinePhiladelphia, PA; 3 Office of Clinical Research, Emory University School of MedicineAtlanta, GA, USA

**Keywords:** sickle cell disease, inflammation, paediatric haematology, vascular biology

## Abstract

Several lines of evidence suggest that sickle cell disease (SCD) is associated with a chronic inflammatory state. In this study of 70 children with SCD at steady state evaluated by a broad panel of biomarkers representing previously examined mechanisms of pathogenicity in SCD, high sensitivity C-reactive protein (hs-CRP), a marker of low-grade, systemic inflammation, emerged as the most significant laboratory correlate of hospitalizations for pain or vaso-occlusive (VOC) events. While markers of increased haemolytic status, endothelial activation and coagulation activation all correlated positively with VOC events by univariate analysis, baseline hs-CRP levels provided the most significant contribution to the association in multiple regression models (22%), and, hs-CRP, along with age, provided the best fit in negative binomial models. These data highlight the clinical relevance of the role of inflammation in paediatric VOC, providing both a rationale for future therapeutic strategies targeting inflammation in microvessel occlusive complications of SCD, and the potential clinical use of hs-CRP as a biomarker in childhood SCD.

Microvessel occlusion, the main pathological process underlying the cardinal clinical manifestation of sickle cell disease (SCD), is frequently termed as vaso-occlusive crisis (VOC) or pain crisis (Stuart & Nagel, 2004). Since the description of SCD as a clinical entity nearly a century ago, investigations into the pathophysiology of VOC have been dynamic and manifold, leading to the delineation today of a complex process, encompassing interactions between sickle red blood cells (RBCs), white blood cells (WBCs), endothelium, plasma proteins, and several other factors (Frenette & Atweh, 2007). Additionally, robust laboratory and animal data have further delineated the roles of hemolysis-related decreased nitric oxide bioavailability (Reiter *et al*, 2002), ischaemia-reperfusion injury (Kaul & Hebbel, 2000), and endothelial activation (Embury *et al*, 2004; Belcher *et al*, 2005) in exacerbating the microvessel occlusive events of SCD.

Several lines of evidence suggest that SCD is associated with a chronic inflammatory state (Platt, 2000; Belcher *et al*, 2003). In recent years there has been great interest in the role of high sensitivity C-reactive protein (hs-CRP) as a stable plasma biomarker of low-grade, chronic, systemic inflammation in predicting risk for cardiovascular disease in adults (Koenig *et al*, 1999; Ridker *et al*, 2000; Verma *et al*, 2005). In this study we evaluate a panel of biomarkers, including hs-CRP, that are representative of previously proposed mechanisms of microvessel occlusion-related clinical events in SCD for their associations with painful episodes in childhood SCD.

We have studied a cohort of children and adolescents with SCD at baseline, to ascertain if biomarker assessments done during the clinically silent ‘steady state’ condition correlate with hospitalization for pain, a signature clinical outcome of microvessel occlusion and indicator of sub-clinical disease burden (Platt *et al*, 1994; Miller *et al*, 2000). Key biomarkers from the panel included: total WBC Count and hs-CRP as markers of systemic global inflammatory activity; Haemoglobin (Hb), and lactate dehydrogenase (LDH) as reflective of haemolytic status (Kato *et al*, 2006);fetal haemoglobin (HbF) level, an indicator of protection against VOC (Powars *et al*, 1989); soluble Vascular Cell Adhesion Molecule-1 (sVCAM1) (Kato *et al*, 2005) and sP-selectin (Burger *et al* 2003; Frenette *et al*, 1998) as indices of endothelial activation; prothrombin fragment F1.2, a marker of thrombin generation, and D-Dimer, a measure of fibrin formation and dissolution. Our aim is to measure these laboratory markers at baseline for an association with the clinical endpoint of acute VOC requiring hospitalization. Our goal is to add to the current state of knowledge regarding the clinical significance of laboratory biomarkers with respect to microvessel occlusive complications of SCD. Iterative validation of such laboratory biomarkers in the clinical setting would also result in establishing objective measures of the success or failure of novel interventions for these complications.

## Methods

### Patient population

The study population included 70 children with SCD (HbSS, HbSβ^0^thalassemia, and HbSC genotype) aged 2–20 years, evaluated in basal steady state at the time of their ‘routine’ health care maintenance visit to the sickle cell clinic. Patients were considered to be in steady state if they were afebrile, asymptomatic with SCD, and had not been hospitalized for at least 10 d prior to date of blood draw. No patient was on chronic transfusion therapy. No subject was on hydroxycarbamide therapy. Age-matched HbAA controls (3–21 years) were also included. This study was reviewed and approved by the Institutional Review Committee for the protection of human subjects at St Christopher’s Hospital for Children/Drexel University and Thomas Jefferson University. In accordance with the Declaration of Helsinki, informed consent was obtained prior to patient blood sampling at the time of enrollment in these studies. For minors, patient assent where appropriate, was obtained in addition to parental permission. A retrospective review was performed on individual patient records for a discharge diagnosis of painful episode or VOC, including those complicated by the occurrence of acute chest syndrome (ACS, chest-wall pain in association with findings of a new pulmonary infiltrate on chest X-ray films and fever (Charache *et al*, 1995). Total painful episodes over a cumulative 3-year period including the calendar year of the blood draw, the year previous to, and the year following date of blood draw were included in the analyses. Blood was drawn by a well-trained phlebotomist using a 2-syringe technique. Blood was collected into appropriate anticoagulant tubes, platelet-poor plasma was prepared by centrifuging whole blood at 1500 ***g*** for 10 min, aliquots of plasma were stored at −70°C.

### Biomarker assays

Biomarkers were assayed by commercially available enzyme-linked immunosorbent assay kits as follows: Those pertinent to inflammation and endothelial activation (hs-CRP-ALPCO Immunoassays Salem, NH, sVCAM1 and sP-selectin; R & D Systems, Minneapolis, MN, USA); and coagulation activation/fibrinolysis (prothrombin fragment F1.2-Enzygnost, Dade Behring Inc. Newark, DE, D-Dimer-Imuclone, American Diagnostica, Stamford, CT). LDH levels were measured using an LDH assay kit (TOX07; Sigma Aldrich, St Louis, MO, USA). The enzyme activity in i/u per litre was read from a calibration curve generated using an LDH standard (Sigma Aldrich). Haematological indices (haemoglobin levels, WBC, and platelet counts) were obtained using a Coulter Counter, Model StK S. Statistics was performed using Sigmastat statistical package (Jandel Scientific, San Rafael, CA, USA). Association between any two variables was tested with Spearman and Kendall Tau Correlation (Conover, 1980). Multiple regression analyses were performed using forward and backward regression models employing rank-transformed data. Further regression analyses were conducted using a negative binomial model in the Statistical Package for the Social Sciences (spss; SPSS Inc., Chicago, IL, USA) for Windows version 15 to account for overdispersion and excessive zeros in the dependent variable, i.e. the number of hospitalizations for pain in the designated 3-year period.

## Results

Seventy plasma samples from SCD children aged 2–20 years were evaluated; HbSS/HbSβ^0^thalassemia *n* = 48 including *n* = 5 with HbSβ^0^thalassemia (henceforth referred to as HbSS group), HbSC, *n* = 22; males = 37, females = 33. Clinical and laboratory data were consistent with previously described haematological parameters in children with SCD [WBC and HbF: HbSS > HbSC; Haemoglobin: HbSS < HbSC (*P* < 0·0001 in each case)] and hospitalizations for pain HbSS > HbSC, *P* = 0·001 (Table I). (Of note, 45% of patients had no episodes of pain, 30% of the patient cohort had >3 total hospitalizations, and 7% had >9 total hospitalizations over the 3-year period). Similarly, baseline values of biomarkers were greater in HbSS patients *versus* HbSC with regard to coagulation activation [F1.2 (*P* = 0·017), DDimer (*P* < 0·001)], haemolysis (LDH) (*P* < 0·001), and endothelial activation [VCAM-1 (*P* = 0·027)], sP-selectin (*P* < 0·001) (Table II). These results were also in keeping with previously published data (Westerman *et al*, 1999; Setty *et al*, 2001, 2003; Mohan *et al*, 2005; Blann *et al*, 2008; O’Driscoll *et al*, 2008).

**Table I:** Haematological data and 3-year cumulative hospitalizations for vaso-occlusive crisis (VOC) data in children with SCD

	Total SCD Group (*n* = 70)	HbSS/HbSβ^0^Thal (*n* = 48)	HbSC (*n* = 22)
HbF (g/l)	74 (35–116)	80 (57–125·5)**	28 (16–42)
Haemoglobin (g/l)	91 (82–107)	85 (79–92)**	111 (105–115)
WBC count (×10^9^/l)	11·3 (8·4–13·1)	12·2 (10·4–14·8)**	7·8 (6·0–9·9)
Platelet count (×10^9^/l)	383 (292–450)	428 (361–524)**	294 (269–347)
Cumulative 3-year hospitalizations for pain	1 (0–4)	1 (0–5)*	0 (0–1)

Values are presented as median (25th–75th) percentiles. Statistical differences in variables between HbSS/HbSβ^0^Thal group and HbSC group are notated as **P < 0·0001 and *P = 0·001 (Mann–Whitney Rank Sum Test). A retrospective review was performed on individual patient records for a discharge diagnosis of painful episode or VOC with and without associated diagnosis of acute chest syndrome (ACS). Painful episodes over a cumulative 3-year period including the calendar year of the blood draw, the year previous to, and the year following date of blood draw were included in the analyses.

**Table II:** Biomarker panel in children with SCD

Biomarker	Total SCD group (*n* = 70)	HbSS/HbSβ^0^Thal (*n* = 48)	HbSC (*n* = 22)	Controls
F1·2 (nmol/l)	1·06 (0·76–1·41)	**1·22 (0·78–1·52)	0·84 (0·70–1·11)	0·73 (0·41–1·27) (*n* = 29)
D-Dimer (ng/ml)	97 (33–209) (*n* = 60)	***168 (88–290) (*n* = 38)	32 (32–80)	48 (32–88) (*n* = 29)
LDH (i/u per litre)	371 (268–504)	***449 (303–619)	272 (240–336)	246 (171–291) (*n* = 29)
sVCAM-1 (ng/ml)	748 (587–1004)	*783 (610–1049)	628 (539–846)	538 (446–628) (*n* = 29)
sP-Selectin (ng/ml)	54 (45–63)	***58 (50–67)	45 (38–50)	40 (33–50) (*n* = 27)
hs-CRP (mg/l)	1·3 (0·6–3·8)	***2·8(1·02–5·3)	0·6 (0·3–1·2)	0·1(0·05–0·5) (*n* = 10)

Results presented are the median (25th–75th) percentile values. Biomarkers from HbSS are significantly different from the respective HbSC levels at *P = 0·027, **P = 0·017 and ***P < 0·001 as assessed by Mann–Whitney Rank Sum Test. Biomarkers from the age-matched HbAA controls are also shown for comparison; these ranges are similar to those previously published from our laboratory and others. (Blann *et al*, 2008 *et al*, 2008; Mohan *et al*, 2005; O’Driscoll *et al*, 2008; Setty *et al*, 2001, 2003; Westerman *et al*, 1999 respectively).

Additionally, we also compared the distribution of hs-CRP values in the study HbSS and HbSC populations to age- and race-matched control ‘reference range’ hs-CRP data available from a large population study (the National Health and Nutritional Examination Study/NHANES –Ford *et al*, 2003). A markedly higher median hs-CRP level was noted in the HbSS group [*n* = 48, median CRP = 2·8 mg/l (1·04–5·6 mg/l, 25th–75th percentile)], whereas median hs-CRP in the HbSC group [*n* = 22, median CRP = 0·6 mg/l (0·3–1·2 mg/l, 25th–75th percentile)] was similar to the NHANES control values [median CRP = 0·4 mg/l (0·1–1·2 mg/l, 25th–75th percentile)] as well as the smaller number of age-matched controls from our study [*n* = 10, median CRP = 0·1 mg/l (0·05–0·5 mg/l, 25th–75th percentile)]. In 25 of the 70 patients, hs-CRP levels were assayed on available plasma samples from at least two different time points (ranging from 3 to 9 months apart). Intra-individual variability for CRP levels in these 25 samples was low, as evidenced by strong association between the pairs of measurements by Kendall Tau rank correlation (τ = 0·69; *P* < 0·0001) and Spearman rank correlation (Spearman *r* = 0·89, *P* < 0·0001; raw data and correlation graphs are available in Table SI).

### Analyses for correlations between biomarkers and VOC related hospitalizations

In the SCD patient group as a whole, by univariate correlation, all values except platelet count, sP-selectin and HbF showed statistically significant correlations with 3-year cumulative hospitalizations for pain as the dependent variable (Table III). In the group of patients with the more severe phenotype, the HbSS group, univariate analysis demonstrated that inflammatory biomarkers (hs-CRP and WBC) remained significantly positively correlated with hospitalizations for pain over the 3-year period. In addition, in accordance with prior studies, HbF levels were ‘protective’, inversely correlating with hospitalizations only in the HbSS group, with the lack of correlation in the total SCD cohort explained by the expected low mean HbF level in HbSC disease. Of note, when hs-CRP values from 25 samples obtained from subjects at a different time point than that used in the initial analysis were substituted and re-analyzed, univariate correlations between 3-year cumulative hospitalizations for pain and hs-CRP levels remained unchanged. In the total SCD group Spearman *r* = 0·532 for substituted CRP values *versus r* = 0·469 for original values; in the HbSS group Spearman *r* = 0·472 for substituted CRP values *versus r* = 0·391 for original values.

**Table III:** Biomarker correlations with 3-year cumulative hospitalizations for vaso-occlusive crises in children with SCD

	Total SCD group (*n* = 70)		HbSS/HbSβ^0^Thal (*n* = 48)		HbSC (*n* = 22)	
			
Biomarker variable	Spearman *r*	*P*	Spearman *r*	*P*	Spearman *r*	*P*
Hb	−0·364	0·002	−0·082	0·58	0·053	0·81
HbF	−0·037	0·75	−0·331	0·02	−0·307	0·16
WBC	0·406	<0·0001	0·286	0·04	−0·127	0·56
Platelets	0·170	0·15	0·036	0·81	−0·463	0·02
LDH	0·379	0·001	0·260	0·07	−0·079	0·72
sVCAM-1	0·282	0·01	0·190	0·19	0·313	0·15
sP-Selectin	0·198	0·1	0·04	0·78	−0·344	0·11
F1·2	0·249	0·03	0·058	0·69	0·341	0·11
D-Dimer*	0·360	0·005	0·09	0·58	0·217	0·32
hs-CRP	0·469	<0·0001	0·391	0·006	0·076	0·73

Correlations were analyzed using Spearman’s rank correlation test.

* D-Dimer values were obtained from 60 of the 70 patients with SCD and 38 of the 48 subjects with HbSS/HbSβ^0^Thal.

Multiple regression analyses (performed in the SCD group as a whole with 3-year cumulative hospitalizations as the dependent variable, and hs-CRP, sP-selectin, VCAM-1, LDH, WBC and HbF as the independent variables) identified associations only with hs-CRP and WBC which contributed 22% and 5% respectively, to the overall model. When data from HbSS group alone were evaluated in the regression models, only hs-CRP stayed in the models contributing 13% to the overall association. These results were confirmed with a negative binomial regression model that identified age and hs-CRP as the best fit to these count data, predicting a 12% increase in pain frequency for every year of age, and a 6·6% increase in pain frequency for every 1 mg/l increase in hs-CRP (Table IV). Thus hs-CRP showed the strongest statistical association between a laboratory biomarker and an important clinical endpoint – increased hospitalizations for pain – highlighting its potential clinical relevance.

**Table IV:** Negative binomial regression analysis

Variable	Parameter estimate exp (B)	95% confidence interval (lower)	95% confidence interval (higher)	Wald Chi-square	*P*-value
Intercept	0·760	0·410	1·410	33·777	<0·001
Age	1·119	1·057	1·184	16·797	<0·001
hs-CRP	1·066	1·001	1·134	3·992	0·046

A negative binomial model was used to account for over-dispersion and excessive zeros in the dependent variable, i.e. the cumulative number of hospitalizations for pain in the designated 3-year period. The goodness of fit of negative binomial models was estimated by log likelihood function. Age and hs-CRP provided the best fit to these count data, predicting a 12% increase in pain frequency for every year of age, and a 6·6% increase in pain frequency for every 1 mg/l increase in hs-CRP.

### Correlations between hs-CRP and other biomarkers evaluated

In the SCD group (Table V), hs-CRP showed an inverse correlation with Hb, and a positive one with LDH, suggesting that baseline haemolytic activity may be associated with inflammation. The correlation of hs-CRP with WBC counts further supported an increase in the baseline inflammation status. In addition, hs-CRP correlated significantly with markers of coagulation activation (F1.2, D-Dimer), and endothelial activation (sVCAM1 and sP-selectin), perhaps underscoring the key role of inflammation in connecting different pathological pathways in SCD. No association between hs-CRP and fetal haemoglobin was noted in the SCD cohort as a whole, while in the HbSS/Sβ^0^Thal patient group the inverse correlation between CRP and HbF levels fell short of statistical significance in this study (*r* = −0·27; *P* = 0·067).

**Table V:** Biomarker correlations with hs-CRP

	Total SCD group (*n* = 70)		HbSS/HbS β^0^Thal (*n* = 48)		HbSC (*n* = 22)	
			
Biomarker variable	Spearman *r*	*P*	Spearman *r*	*P*	Spearman *r*	*P*
Hb	−0·56	<0·0001	−0·28	0·057	−0·42	0·05
HbF	0·10	0·41	−0·27	0·067	0·08	0·70
LDH	0·40	<0·0001	0·25	0·084	0·05	0·82
Platelets	0·27	0·025	0·02	0·912	−0·04	0·84
sVCAM-1	0·29	0·014	0·22	0·135	0·16	0·46
P-Selectin	0·48	<0·001	0·33	0·02	0·17	0·43
F1·2	0·43	<0·001	0·33	0·022	0·32	0·15
D-Dimer*	0·63	<0·001	0·47	0·003	0·46	0·02
WBC	0·46	<0·001	0·21	0·157	0·18	0·43

Correlations were analyzed using Spearman’s rank correlation test.

* D-Dimer data was obtained from 60 of the 70 patients with SCD and 38 of the 48 subjects with HbSS/HbSβ^0^Thal.

## Discussion

This study of children and adolescents with SCD showed that, from a broad panel of steady-state biomarkers, the inflammatory marker hs-CRP was the most significant correlate of hospitalizations for painful episodes. While markers of increased haemolytic status, endothelial activation and coagulation activation all correlated positively with VOC events by univariate analysis, only the inflammatory markers hs-CRP and WBC stayed in multiple regression models, and age and hs-CRP provided the best fit in negative binominal models. These data showed that hs-CRP, a well- established inflammatory biomarker (Pepys & Hirschfield, 2003), was strongly associated with paediatric VOC, an important clinical endpoint of microvessel occlusion in SCD.

Over recent years, the role of hs-CRP as a plasma biomarker for low-grade systemic inflammation has been intensely investigated for its predictive associations with adverse outcomes in vascular diseases, such as cardiovascular (Cook *et al*, 2006; Folsom *et al*, 2006) and peripheral arterial disease (Vainas *et al*, 2005; Vidula *et al*, 2008). The pentatraxin protein CRP is produced in the liver as part of the acute phase reaction, in response to a host of pro-inflammatory cytokines (Hurlimann *et al*, 1966; Moshage *et al*, 1988). Circulating CRP levels are determined solely by its rate of synthesis and thus reflect the presence and strength of pathological stimuli that are present in the individual at the time of evaluation (Vigushin *et al*, 1993). Barring elevations in CRP that are associated with acute infections and inflammation, CRP concentrations are generally stable, falling within a characteristic range for each individual (Macy *et al*, 1997; Pepys & Hirschfield, 2003). A subset analysis of 25 patients showed that we have reason to believe that SCD patients evaluated at steady state will also demonstrate intra-individual stability of hs-CRP levels, which would be reflective of their baseline state of inflammatory response. CRP is exceptionally stable in serum or plasma when stored at −70°C and readily available immunoassays have been well validated in population studies (Kimberly *et al*, 2003; Roberts, 2004). While our results from a retrospective cohort study need to be confirmed in a prospective manner, the advantages of studying a well-validated biomarker, such as hs-CRP, in SCD are manifold.

Many studies have documented altered pro-inflammatory cytokine levels in the plasma of SCD patients during both steady-state and acute vaso-occlusive crisis (Bourantas *et al*, 1998; Duits *et al*, 1998; Pathare *et al*, 2003), but no consistent pattern of cytokines involvement in SCD has emerged that correlates with specific clinical outcomes. However, these cytokines, in turn, could be responsible for driving the low-grade or chronic inflammatory response, evidenced by the presence of mild-moderate, baseline elevations of acute phase reactants, such as CRP (Singhal *et al*, 1993). Two paediatric studies found increases in baseline hs-CRP levels that correlate with increased resting energy expenditure or the ‘hypermetabolic’ state in SCD (*n* = 12) (Hibbert *et al*, 2005), and oxidant stress (*n* = 35) (Akohoue *et al*, 2007). Clinical data from adult SCD patients also document baseline CRP elevations (Mohan *et al*, 2005). In one study, baseline elevations in CRP in adult HbSS/Sβ^0^Thalassemia patients were correlated with a ‘sickle severity index’ as compared to heterozygous sickle beta thalassemia patients (Hedo *et al*, 1993; Makis *et al*, 2006). Taken together, these studies documenting steady-state CRP elevations suggest the presence of an ongoing inflammatory response during symptom-free ‘steady state’ periods. To our knowledge, no data is available with respect to the association of chronic inflammation with specific clinical outcomes in paediatric SCD.

In this context, our retrospective study, involving a moderate-sized paediatric SCD cohort and covering the age spectrum of 2–20 years, provides strong support for the association of chronic inflammation with acute VOC/pain episodes. In the study cohort, baseline median hs-CRP levels were increased compared to population-defined age- and race-matched control levels. More significantly, hs-CRP levels showed a strong statistical association by appropriate regression modelling procedures with an important clinical endpoint – increased hospitalizations for pain. Typically, patients with homozygous HbSS/HbSβ^0^Thal manifest the highest rate of vasocclusive events, as corroborated by our findings. Our data show that children with the more severe phenotype, HbSS/HbSβ^0^Thalassemia, have significantly higher baseline hs-CRP levels than those with HbSC disease. This finding suggests that the presence of ongoing ‘significant subclinical’ inflammation in HbSS/HbSβ^0^Thal patients perhaps puts them at greater risk for experiencing acute vasocclusive events.

In the HbSS group (Table III), inflammatory markers hs-CRP and WBC, were strongly associated with clinical outcome of hospitalization for pain events, while markers of activation of coagulation (F1.2, D-Dimer), and endothelial activation (sVCAM1 and sP-selectin) were not associated with the clinical outcome of hospitalization for pain events. However, in the same patient subgroup (Table V), hs-CRP showed excellent correlation with these latter markers (F1.2, D-Dimer, and sP-selectin) perhaps underscoring the intersection of inflammation with multiple pathological pathways in SCD. It is possible that some of these laboratory markers of additional putative pathogenic pathways of SCD microvessel occlusion are not as robust in clinical analyses because of assay-related or phenomenological reasons. We suggest that our data indicate the strength of hs-CRP as a laboratory tool to assess not only chronic inflammation, but also, via its associations with markers of other pathogenic pathways, to reflect underlying endothelial and coagulation activation as well, thus making hs-CRP a more biologically relevant marker of SCD-related activity.

We also determined correlations of hs-CRP with other biomarkers in the panel evaluated and found that hs-CRP was not associated with fetal haemoglobin levels in our study cohort (SCD *r* = 0·1, *P* = 0·41; HbSS/HbS/beta^0^Thalassemia *r* = −0·27, *P* = 0·067 –Table V). Overall, we found no statistically significant correlation between hs-CRP and markers of hemolysis (LDH and Hb) in the HbSS group. Lower HbF levels are known to be associated with higher pain rates (Platt *et al*, 1994), and provide the rationale for the use of HbF modulators to treat SCD-related pain. However, in the Multi-Center Study of Hydroxycarbamide, Charache *et al* (1995), noted that some patients had a clinical response even before sustained elevation of HbF implying that HbF modifiers like hydroxycarbamide can affect sickle cell anaemia by mechanisms other than increase in fetal haemoglobin. We submit that the lack of a clear association between HbF and hs-CRP in our study perhaps gives credence to the involvement of additional pathogenic mechanisms, such as inflammation, in SCD vaso-occlusion.

In addition to being a retrospective analysis there are other limitations to our study, which we have tried to address. Though we do not have longitudinal data on the entire study sample, in a subset analysis of 25 patients with two baseline or steady state measurements ranging from 3 to 9 months apart, we found strong short-term intra-individual correlation between the two sample measurements. This provides us with some basis to believe that SCD patients will also demonstrate longitudinal stability of hs-CRP levels, though this needs to be confirmed in a larger sample. We have used a 3-year cumulative hospitalization for pain centered upon the year that plasma biomarker determinations were done. This is because the frequency of pain hospitalizations, while relatively consistent from year to year, is infrequent in any given year. An overdispersed negative binomial model was used to confirm multiple regression results because the dependent variable consisted of discrete hospitalization events that resulted in sparse count data (excessive zeroes). Our cohort was also skewed more toward younger children given our interest in the development of SCD symptomatology from early childhood through adolescence and young adulthood. However, we feel that because of this unique data set, we have been able to document hs-CRP elevations at a relatively young age in the HbSS with potential future clinical consequences.

In summary, the present study demonstrated a strong association between the inflammatory biomarker hs-CRP and hospitalization for VOC events in paediatric SCD. We believe these results highlight the clinical relevance of inflammation in microvessel occlusive complications, and provide a basis for the study of hs-CRP as a potential biomarker for predictive modelling of clinical outcomes in paediatric SCD. Perhaps most significantly, these data culled from a moderate-sized cohort of 70 children with SCD, lend support for the clinical reconciliation of strong experimental evidence for the role of inflammation in SCD vasoocclusion, and provide a basis for the future trials of primary or adjuvant anti-inflammatory therapies in SCD.

## Supporting information

**Table SI.** Intra-individual variability of hs-CRP levels.
