# Supplementary material for: Increased levels of the inflammatory biomarker C-reactive protein at baseline are associated with childhood sickle cell vasocclusive crises
Source: Br J Haematol. 2010 Mar;148(5):797–804. doi: 10.1111/j.1365-2141.2009.08013.x (PMC2878774; doi:10.1111/j.1365-2141.2009.08013.x)
Supplement: Supplementary file 1 [file bjh0148-0797-SD1.doc]

**Table: Intra-individual Variability of hs-CRP levels**

|  | **CRP (mg/L)** | |
| --- | --- | --- |
|  | Time Point #1 | Time Point # 2 |
| Subject #1 | 0.4 | 0.02 |
| Subject #2 | 2.6 | 2.7 |
| Subject #3 | 0.1 | 0.2 |
| Subject #4 | 4.0 | 2.7 |
| Subject #5 | 1.1 | 2.5 |
| Subject #6 | 0.1 | 0.2 |
| Subject #7 | 1.0 | 0.9 |
| Subject #8 | 2.2 | 4.9 |
| Subject #9 | 5.9 | 5.9 |
| Subject #10 | 0.7 | 1.9 |
| Subject #11 | 0.5 | 0.3 |
| Subject #12 | 6.8 | 3.5 |
| Subject #13 | 0.2 | 0.3 |
| Subject #14 | 2.7 | 2.3 |
| Subject #15 | 4.4 | 3.8 |
| Subject #16 | 25.1 | 3.9 |
| Subject #17 | 0.5 | 0.5 |
| Subject #18 | 0.05 | 0.3 |
| Subject #19 | 0.1 | 0.04 |
| Subject #20 | 0.1 | 0.2 |
| Subject #21 | 0.2 | 0.3 |
| Subject #22 | 2.8 | 4.6 |
| Subject #23 | 2.2 | 0.4 |
| Subject #24 | 4.6 | 11.9 |
| Subject #25 | 0.5 | 1.5 |

hs-CRP measurements from two different time points (ranging from 3-9 months apart) available for 25 subjects are presented in this table.

Of note, risk stratification for Cardiovascular disease based on CRP levels is as follows: <1mg/L= Low risk, 1 to 2.9 mg/L = Intermediate risk, and >3mg/L= High risk. These values are presented to contextualize the stability or change in CRP level status of the individual subject from Time Point #1 to Time Point #2.

**Spearman Correlation between hs-CRP levels measured at two different times**: Values presented are the rank-transformed hs-CRP levels from 25 donors measured at two different times (3 to 9 months apart). The solid, dash, and dash-dot-dot lines represent the regression, and the 95% and 99% confidence interval lines.
